# Overview of Virus Metagenomic Classification Methods and Their Biological Applications

**DOI:** 10.3389/fmicb.2018.00749

**Published:** 2018-04-23

**Authors:** Sam Nooij, Dennis Schmitz, Harry Vennema, Annelies Kroneman, Marion P. G. Koopmans

**Affiliations:** ^1^Emerging and Endemic Viruses, Centre for Infectious Disease Control, National Institute for Public Health and the Environment (RIVM), Bilthoven, Netherlands; ^2^Viroscience Laboratory, Erasmus University Medical Centre, Rotterdam, Netherlands

**Keywords:** pipeline, decision tree, software, use case, standardization, viral metagenomics

## Abstract

Metagenomics poses opportunities for clinical and public health virology applications by offering a way to assess complete taxonomic composition of a clinical sample in an unbiased way. However, the techniques required are complicated and analysis standards have yet to develop. This, together with the wealth of different tools and workflows that have been proposed, poses a barrier for new users. We evaluated 49 published computational classification workflows for virus metagenomics in a literature review. To this end, we described the methods of existing workflows by breaking them up into five general steps and assessed their ease-of-use and validation experiments. Performance scores of previous benchmarks were summarized and correlations between methods and performance were investigated. We indicate the potential suitability of the different workflows for (1) time-constrained diagnostics, (2) surveillance and outbreak source tracing, (3) detection of remote homologies (discovery), and (4) biodiversity studies. We provide two decision trees for virologists to help select a workflow for medical or biodiversity studies, as well as directions for future developments in clinical viral metagenomics.

## Introduction

Unbiased sequencing of nucleic acids from environmental samples has great potential for the discovery and identification of diverse microorganisms (Tang and Chiu, 2010; Chiu, 2013; Culligan et al., 2014; Pallen, 2014). We know this technique as metagenomics, or random, agnostic or shotgun high-throughput sequencing. In theory, metagenomics techniques enable the identification and genomic characterisation of all microorganisms present in a sample with a generic lab procedure (Wooley and Ye, 2009). The approach has gained popularity with the introduction of next-generation sequencing (NGS) methods that provide more data in less time at a lower cost than previous sequencing techniques. While initially mainly applied to the analysis of the bacterial diversity, modifications in sample preparation protocols allowed characterisation of viral genomes as well. The fields of virus discovery and biodiversity characterisation have seized the opportunity to expand their knowledge (Cardenas and Tiedje, 2008; Tang and Chiu, 2010; Chiu, 2013; Pallen, 2014).

There is interest among virology researchers to explore the use of metagenomics techniques, in particular as a catch-all for viruses that cannot be cultured (Yozwiak et al., 2012; Smits and Osterhaus, 2013; Byrd et al., 2014; Naccache et al., 2014; Pallen, 2014; Smits et al., 2015; Graf et al., 2016). Metagenomics can also be used to benefit patients with uncommon disease etiologies that otherwise require multiple targeted tests to resolve (Chiu, 2013; Pallen, 2014). However, implementation of metagenomics in the routine clinical and public health research still faces challenges, because clinical application requires standardized, validated wet-lab procedures, meeting requirements compatible with accreditation demands (Hall et al., 2015). Another barrier is the requirement of appropriate bioinformatics analysis of the datasets generated. Here, we review computational workflows for data analysis from a user perspective.

Translating NGS outputs into clinically or biologically relevant information requires robust classification of sequence reads—the classical “what is there?” question of metagenomics. With previous sequencing methods, sequences were typically classified by NCBI BLAST (Altschul et al., 1990) against the NCBI nt database (NCBI, 2017). With NGS, however, the analysis needs to handle much larger quantities of short (up to 300 bp) reads for which proper references are not always available and take into account possible sequencing errors made by the machine. Therefore, NGS needs specialized analysis methods. Many bioinformaticians have developed computational workflows to analyse viral metagenomes. Their publications describe a range of computer tools for taxonomic classification. Although these tools can be useful, selecting the appropriate workflow can be difficult, especially for the computationally less-experienced user (Posada-Cespedes et al., 2016; Rose et al., 2016).

A part of the metagenomics workflows has been tested and described in review articles (Bazinet and Cummings, 2012; Garcia-Etxebarria et al., 2014; Peabody et al., 2015; Sharma et al., 2015; Lindgreen et al., 2016; Posada-Cespedes et al., 2016; Rose et al., 2016; Sangwan et al., 2016; Tangherlini et al., 2016) and on websites of projects that collect, describe, compare and test metagenomics analysis tools (Henry et al., 2014; CAMI, 2016; ELIXIR, 2016). Some of these studies involve benchmark tests of a selection of tools, while others provide brief descriptions. Also, when a new pipeline is published the authors often compare it to its main competitors. Such tests are invaluable to assessing the performance and they help create insight into which tool is applicable to which type of study.

We present an overview and critical appraisal of available virus metagenomic classification tools and present guidelines for virologists to select a workflow suitable for their studies by (1) listing available methods, (2) describing how the methods work, (3) assessing how well these methods perform by summarizing previous benchmarks, and (4) listing for which purposes they can be used. To this end, we reviewed publications describing 49 different virus classification tools and workflows—collectively referred to as workflows—that have been published since 2010.

## Methods

We searched literature in PubMed and Google Scholar on classification methods for virus metagenomics data, using the terms “virus metagenomics” and “viral metagenomics.” The results were limited to publications between January 2010 and January 2017. We assessed the workflows with regard to technical characteristics: algorithms used, reference databases, and search strategy used; their user-friendliness: whether a graphical user interface is provided, whether results are visualized, approximate runtime, accepted data types, the type of computer that was used to test the software and the operating system, availability and licensing, and provision of a user manual. In addition, we extracted information that supports the validity of the workflow: tests by the developers, wet-lab experimental work and computational benchmarks, benchmark tests by other groups, whether and when the software had been updated as of 19 July 2017 and the number of citations in Google Scholar as of 28 March 2017 (Data Sheet 1; https://compare.cbs.dtu.dk/inventory#pipeline). We listed only benchmark results from *in silico* tests using simulated viral sequence reads, and only sensitivity, specificity and precision, because these were most often reported (Data Sheet 2). Sensitivity is defined as reads correctly annotated as viral—on the taxonomic level chosen in that benchmark—by the pipeline as a fraction of the total number of simulated viral reads (true positives / (true positives + false negatives)). Specificity as reads correctly annotated as non-viral by the pipeline as a fraction of the total number of simulated non-viral reads (true negatives / (true negatives + false positives)). And precision as the reads correctly annotated as viral by the pipeline as a fraction of all reads annotated as viral (true positives / (true positives + false positives)). Different publications have used different taxonomic levels for classification, from kingdom to species. We used all benchmark scores for our analyses (details are in Data Sheet 2). Correlations between performance (sensitivity, specificity, precision and runtime) and methodical factors (different analysis steps, search algorithms and reference databases) were calculated and visualized with R v3.3.2 (https://www.r-project.org/), using RStudio v1.0.136 (https://www.rstudio.com).

Next, based on our inventory, we grouped workflows by compiling two decision trees to help readers select a workflow applicable to their research. We defined “time-restrained diagnostics” as being able to detect viruses and classify to genus or species in under 5 h per sample. “Surveillance and outbreak tracing” refers to the ability of more specific identification to the subspecies-level (e.g., genotype). “Discovery” refers to the ability to detect remote homologs by using a reference database that covers a wide range of viral taxa combined with a sensitive search algorithm, i.e., amino acid (protein) alignment or composition search. For “biodiversity studies” we qualified all workflows that can classify different viruses (i.e., are not focused on a single species).

Figures were made with Microsoft PowerPoint and Visio 2010 (v14.0.7181.5000, 32-bit; Redmond, Washington, U.S.A.), R packages pheatmap v1.0.8 and ggplot2 v2.2.1, and GNU Image Manipulation Program (GIMP; v2.8.22; https://www.gimp.org).

## Results and workflow descriptions

### Available workflows

We found 56 publications describing the development and testing of 49 classification workflows, of which three were unavailable for download or online use and two were only available upon request (Table 1). Among these were 24 virus-specific workflows, while 25 were developed for broader use, such as classification of bacteria and archaea. The information of the unavailable workflows has been summarized, but they were not included in the decision trees. An overview of all publications, workflows and scoring criteria is available in Data Sheet 1 and on https://compare.cbs.dtu.dk/inventory#pipeline.

**Table 1:** Classification workflows and their reference.

**Name**	**References**	**URL**
CaPSID	Borozan et al., 2012	https://github.com/capsid/capsid
ClassyFlu	Van der Auwera et al., 2014	http://bioinf.uni-greifswald.de/ClassyFlu/query/init
Clinical PathoScope	Byrd et al., 2014	https://sourceforge.net/p/pathoscope/wiki/clinical_pathoscope/
DUDes	Piro et al., 2016	http://sf.net/p/dudes
EnsembleAssembler	Deng et al., 2015	https://github.com/xutaodeng/EnsembleAssembler
Exhaustive Iterative Assembly (Virus Discovery Pipeline)	Schürch et al., 2014	–
FACS	Stranneheim et al., 2010	https://github.com/SciLifeLab/facs
GenSeed-HMM	Alves et al., 2016	https://sourceforge.net/projects/genseedhmm/
Giant Virus Finder	Kerepesi and Grolmusz, 2016	http://pitgroup.org/giant-virus-finder
GOTTCHA	Freitas et al., 2015	https://github.com/LANL-Bioinformatics/GOTTCHA
IMSA	Dimon et al., 2013	https://sourceforge.net/projects/arron-imsa/?source=directory
IMSA+A	Cox et al., 2017	https://github.com/JeremyCoxBMI/IMSA-A
Kraken	Wood and Salzberg, 2014	https://github.com/DerrickWood/kraken
LMAT	Ames et al., 2013	https://sourceforge.net/projects/lmat/
MEGAN 4	Huson et al., 2011	http://ab.inf.uni-tuebingen.de/software/megan4/
MEGAN Community Edition	Huson et al., 2016	http://ab.inf.uni-tuebingen.de/data/software/megan6/download/welcome.html
MePIC	Takeuchi et al., 2014	https://mepic.nih.go.jp/
MetaShot	Fosso et al., 2017	https://github.com/bfosso/MetaShot
metaViC	Modha, 2016	https://github.com/sejmodha/metaViC
Metavir	Roux et al., 2011	http://metavir-meb.univ-bpclermont.fr/
Metavir 2	Roux et al., 2014	http://metavir-meb.univ-bpclermont.fr/
MetLab	Norling et al., 2016	https://github.com/norling/metlab
NBC	Rosen et al., 2011	http://nbc.ece.drexel.edu/
PathSeq	Kostic et al., 2011	https://www.broadinstitute.org/software/pathseq/
ProViDE	Ghosh et al., 2011	http://metagenomics.atc.tcs.com/binning/ProViDE/
QuasQ	Poh et al., 2013	http://www.statgen.nus.edu.sg/~software/quasq.html
READSCAN	Naeem et al., 2013	http://cbrc.kaust.edu.sa/readscan/
Rega Typing Tool	Kroneman et al., 2011; Pineda-Peña et al., 2013	http://regatools.med.kuleuven.be/typing/v3/hiv/typingtool/
RIEMS	Scheuch et al., 2015	https://www.fli.de/fileadmin/FLI/IVD/Microarray-Diagnostics/RIEMS.tar.gz
RINS	Bhaduri et al., 2012	http://khavarilab.stanford.edu/tools-1/#tools
SLIM	Cotten et al., 2014	“Available upon request”
SMART	Lee et al., 2016	https://bitbucket.org/ayl/smart
SRSA	Isakov et al., 2011	“Available upon request”
SURPI	Naccache et al., 2014	https://github.com/chiulab/surpi
Taxonomer	Flygare et al., 2016	https://www.taxonomer.com/
Taxy-Pro	Klingenberg et al., 2013	http://gobics.de/TaxyPro/
“Unknown pathogens from mixed clinical samples”	Gong et al., 2016	–
vFam	Skewes-Cox et al., 2014	http://derisilab.ucsf.edu/software/vFam/
VIP	Li et al., 2016	https://github.com/keylabivdc/VIP
ViralFusionSeq	Li et al., 2013	https://sourceforge.net/projects/viralfusionseq/
Virana	Schelhorn et al., 2013	https://github.com/schelhorn/virana
VirFind	Ho and Tzanetakis, 2014	http://virfind.org/j/
VIROME	Wommack et al., 2012	http://virome.dbi.udel.edu/app/#view=home
ViromeScan	Rampelli et al., 2016	https://sourceforge.net/projects/viromescan/
VirSorter	Roux et al., 2015	https://github.com/simroux/VirSorter
VirusFinder	Wang et al., 2013	http://bioinfo.mc.vanderbilt.edu/VirusFinder/
VirusHunter	Zhao et al., 2013	https://www.ibridgenetwork.org/#!/profiles/9055559575893/innovations/103/
VirusSeeker	Zhao et al., 2017	https://wupathlabs.wustl.edu/virusseeker/
VirusSeq	Chen et al., 2013	http://odin.mdacc.tmc.edu/~xsu1/VirusSeq.html
VirVarSeq	Verbist et al., 2015	http://sourceforge.net/projects/virtools/?source=directory
VMGAP	Lorenzi et al., 2011	–

–*, No website could be found, the workflow was unavailable*.

### Metagenomics classification methods

**Figure 1:**
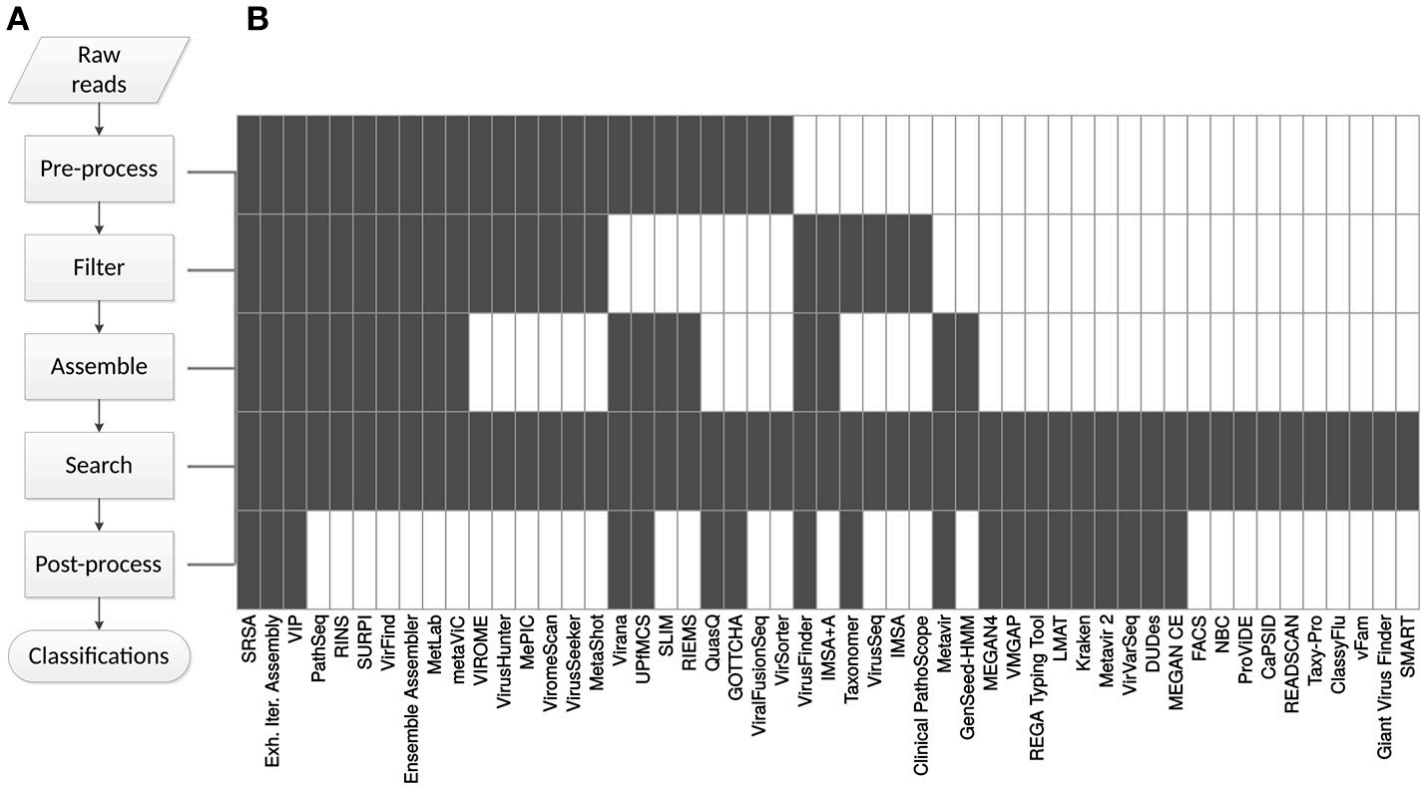
Generic pipeline scheme and breakdown of tools. **(A)** The process of classifying raw sequencing reads in 5 generic steps. **(B)** The steps that workflows use (in gray). UPfMCS: “Unknown Pathogens from Mixed Clinical Samples”; MEGAN CE: MEGAN Community Edition.

The selected metagenomics classification workflows consist of up to five different steps: pre-process, filter, assembly, search and post-process (Figure 1A). Only three workflows (SRSA, Isakov et al., 2011, Exhaustive Iterative Assembly, Schürch et al., 2014, and VIP, Li et al., 2016) incorporated all of these steps. All workflows minimally included a “search” step (Figure 1B, **Table 4**), as this was an inclusion criterion. The order in which the steps are performed varies between workflows and in some workflows steps are performed multiple times. Workflows are often combinations of existing (open source) software, while sometimes, custom solutions are made.

### Quality control and pre-processing

A major determinant for the success of a workflow is the quality of the input reads. Thus, the first step is to assess the data quality and exclude technical errors from further analysis. This may consist of several processes, depending on the sequencing method and demands such as sensitivity and time constraints. The pre-processing may include: removing adapter sequences, trimming low quality reads to a set quality score, removing low quality reads—defined by a low mean or median Phred score assigned by the sequencing machine—removing low complexity reads (nucleotide repeats), removing short reads, deduplication, matching paired-end reads (or removing unmated reads) and removing reads that contain Ns (unresolved nucleotides). The adapters, quality, paired-end reads and accuracy of repeats depend on the sequencing technology. Quality cutoffs for removal are chosen in a trade-off between sensitivity and time constraints: removing reads may result in not finding rare viruses, while having fewer reads to process will speed up the analysis. Twenty-four workflows include a pre-processing step, applying at least one of the components listed above (Figure 1B, Table 2). Other workflows require input of reads pre-processed elsewhere.

**Table 2:** Technical details of classification workflows.

**Workflow**	**Search software**	**Search database**	**Filter software**	**Filter database**	**Assembly software**	**Analysis steps**	**All software used**
CaPSID	Novoalign/Bowtie2 (any)	NCBI Genomes: GenBank viral, bacteria and fungi or custom	Same as search	NCBI human GRCh37/hg19 or custom	–	S	Python 2.7, MongoDB, OpenJDK, BioPython, pysam, Novoalign, BioScope, JBrowse, Groovy-Grails
ClassyFlu	HMMER	NCBI Influenza Virus Resource	–	–	–	S	HMMER
Clinical PathoScope	Bowtie2	NCBI Genomes: viral, bacteria	Bowtie2	NCBI human GRCh37/hg19, GenBank human rRNA	–	SFS	Python, Bowtie2
DUDes	Bowtie2	DUDesDB	–	–	–	S-pp	Python, Bowtie2
EnsembleAssembler	Megablast	NCBI RefSeq: viral, bacteria	Bowtie2	NCBI human GRCh37/hg19	SOAPDenovo, ABySS, MetaVelvet, CAP3	FPAS	Python, SOAPDenovo, ABySS, MetaVelvet, CAP3, MegaBLAST, Bowtie2, VecScreen
Exhaustive Iterative Assembly (Virus Discovery Pipeline)	BLAST, MAFFT, PhyML	NCBI BLAST nt + nr, NCBI viral proteins (per hit), Pfam, custom	BLASTn	NCBI nt: aves, carnivora, primates, rodentia, ruminantia	Newbler, CAP3	PAFSPh	Python, Newbler, GSMapper, CAP3, BLAST, HMMER, MEME, MAST, Bioconductor, R
FACS	Bloom filter	Custom	–	–	–	S	Perl; C
GenSeed–HMM	BLASTx	*Microviridae* proteins (Roux et al., 2012)	–	–	CAP3, Velvet, Newbler, SOAPDenovo, ABySS	AS	HMMER, BLAST, EMBOSS, CAP3, Velvet, Newbler, SOAPDenovo, ABySS
Giant Virus Finder	BLAST	NCBI BLAST nt, custom reference genomes	–	–	–	S	Perl, Python, BLAST
GOTTCHA	BWA (mem)	Custom	–	–	–	PS-pp	Perl, BWA
IMSA	BLAST	Custom	Bowtie, BLAT, BLAST	User-defined (human genome)	–	FS	Python, Blast, Bowtie2, Blat
IMSA+A	Bowtie2	“The reference genome”	–	–	Oases, Velvet, Trinity	FAS	Python, IMSA, BLAST+, BLAT, Bowtie2, Oases, Velvet, Trinity
Kraken	Kraken	NCBI RefSeq: bacteria, NCBI Genomes: GenBank bacteria + archaea	–	–	–	S-pp	C++, Perl
LMAT	Custom	NCBI Genomes: GenBank bacteria	–	–	–	S	gcc, Python, OpenMP, MPI
MEGAN CE	DIAMOND	NCBI BLAST nt	–	–	–	S-pp	Java, DIAMOND, InterPro2GO, SEED viewer, eggNOG viewer, KEGG
MEGAN4	BLAST	NCBI BLAST nt + nr	–	–	–	S	Java
MePIC	Megablast	NCBI BLAST nt	BWA	NCBI human GRCh37/hg19	–	PFS	fastq-mcf (ea-utils), BWA, Megablast
MetaShot	Bowtie2, TANGO	NCBI GenBank: viral, bacteria fungi (from plantae), protitsta (from invertebrate), and NCBI RefSeq: viral, bacteria, fungi, Protista (from invertebrate)	STAR	NCBI human GRCh37/hg19, 2009	–	PFS	Python, Bash, FaQCs, STAR, Bowtie2, TANGO
metaViC	DIAMOND	NCBI RefSeq: Complete protein	RiboPicker	–	IDBA-UD, SPAdes	PFSAS	Bowtie2, DIAMOND, filter_fastq.pl, GARM, IDBA-UD, Kronatools, prinseq, QUAST, riboPicker, SPAdes, Trim Galore
Metavir	BLASTx, MUSCLE, PhyML, HMMER	NCBI RefSeq: viral, Pfam, NCBI BLAST nr	–	–	CAP3	SASPh	MUSCLE, CAP3, Gblocks, PhyML, Scriptree
Metavir 2	BLAST, custom, FastTree	NCBI RefSeq: viral, Pfam	–	–	–	SPh	Perl, Php, Javascript, Css, R, BLAST, FastTree, MetaGeneAnnotator, HMMScan, Uclust, jackhmmer, RaphaelSVG, Cytoscope-web
MetLab	Kraken, HMMER3	NCBI RefSeq: bacteria, archaea, NCBI GenBank: viral, phage, vFams	Bowtie2	“Host genome” (human)	SPAdes	P(F)(A)SS	Python (2.7) + libraries, GNU MPFR, Prinseq-Lite, Bowtie2, SAMTOOLS, SPAdes, Krona Tools, Kraken, FragGeneScan, HMMsearch, vFamParse
NBC	Custom	“Unique N-mer frequency profiles of 635 microbial genomes”	–	–	–	S	Perl, C++
PathSeq	BLAST	NCBI BLAST nt: viruses, gungi, NCBI Genomes: bacteria, NCBI BLAST nr	MAQ, Megablast, BLASTN	1000 Genomes Project: female reference, Ensembl: Homo sapiens cDNA, NCBI BLAST: human genome, transcriptome, NCBI human hs_alt_Celera, hs_alt_HuRef, hs_ref_GRC37, NCBI Genomes: Homo sapiens RNA, Ensembl Homo sapiens DNA	Velvet	PFFAS	Python, Java, C++, C, Hadoop, MAQ, Megablast, Blast, Velvet, RepeatMasker
ProViDE	BLASTx	NCBI BLAST nr	–	–	–	S	Perl, Python
QuasQ	Bowtie2	“The reference genome”	–	–	–	PS-pp	Perl, Bowtie2
READSCAN	SMALT	?	SMALT	?	–	S	Perl, SMALT, Makeflow
Rega Typing Tool v3	BLAST, TreePuzzle	Custom	–	–	–	SPh	Php, Java, R, TreePuzzle
RIEMS	GS Mapper, BLAST	NCBI BLAST nt, nr	–	–	Newbler	PASAS(SS)	Bash, 454 genome sequencer software suite, Newbler, GS mapper, sff/fna tools, BLAST, Emboss
RINS	BLAT, BLAST	Custom	Bowtie	Human genome	Trinity	SPFAS	Blat, Bowtie, Trinity, BLAST
SLIM	BLAST	NCBI GenBank entries of 2,000–500,000 bp long	–	–	SPAdes	PAS	Python, BLAST, QUASR, MEGAN, BWA, SPAdes, MUMmer
SMART	Custom	NCBI GenBank: release v209	–	–	–	S	C++, Ruby, Flash, Sickle, Google SparseHash, GNU parallel
SRSA	Megablast	NCBI GenBank: CDS translations, PDB, SwissProt, PIR, PRF	BWA	NCBI human GRCh37/hg19	Velvet	PFAS	BWA, fastx_toolkit, BLAST, MegaBlast, Velvet
SURPI	RAPSearch2, SNAP	“fast”: NCBI RefSeq: bacteria genomic, NCBI BLAST nt + nr - viridae; “comprehensive”: NCBI BLAST nt, nr,	SNAP	NCBI human GRCh37/hg19, NCBI RefSeq rRNA, mRNA, mtRNA (March 2012)	Minimo, ABySS	PFS(AS)	Bash, Python, Perl, fastQValidator, Minomo, ABySS, RAPSearch2, seqtk, SNAP, gt-sequniq, fastq, cutadapt, prinseq-lite, dropcache
Taxonomer	KAnalyze + custom k-mer matching	UniRef90 viruses	KAnalyze	Greengenes, UNITE, UniRef50, Ensembl	–	FFSS-pp	Cython, KAnalyze
Taxy-Pro	CoMet	Pfam + metagenomes	–	–	–	S	MATLAB, CoMet webserver
“Unknown pathogens from mixed clinical samples”	BLAST	NCBI BLAST nt	–	–	CLC Genomics	SAPh-pp	CLC Genomics Workbench (7.5), Blast, Bowtie2, Clustal Omega, MEGA6, SimPlot
vFam	HMMER	NCBI RefSeq: viral protein	–	–	–	S	HMMER(, CD-HIT, MCL, MUSCLE, BLAST)
VIP	Bowtie2, RAPSearch2 (depending on mode), MAFFT, ETE	“fast”: ViPR/IRD nucleotide DB; “sense”: NCBI RefSeq: viral genomic, viral protein, NCBI GenBank viral neighbor genomes	Bowtie2	NCBI human GRCh38/hg38, NCBI RefSeq rRNA, RNA, mtDNA (July 2015), GOTTCHA bacterial DB	Velvet-Oases	PFSAPh	Shell, Python, Perl, PICARD, Bowtie2, MAFFT, Velvet-Oases, RAPSearch2, ETE
ViralFusionSeq	BWA, BLAST	“Viral sequences and human decoy sequences“	–	–	–	PS	Perl, BWA, BLAST
Virana	STAR(, BLAST, LASTZ)	NCBI RefSeq: “viruses,” Repbase human endogenous retroviruses	STAR	NCBI GRCh37/hg19, Ensembl human cDNA	Trinity, Oases	PS(A)(S)Ph	Python, STAR, BWA-mem, LASTZ, RazerS3, Jalview, Trinity, Oases
VirFind	BLAST	NCBI BLAST nt, NCBI RefSeq viral protein	Bowtie2	Custom	Velvet, CAP3	PFAS(S)	fastx-toolkit, seq_crumbs, Bowtie2, Velvet, Blast, CAP3, Python
VIROME	BLAST	UniRef100, SEED, ACLAME, COG, GO, KEGG, MGOL, CAMERA, UniVec	BLASTn. tRNAscan-SE	”A rRNA subject database“	–	PFS	Adobe Flex, MySQL, Blast, tRNA scan SE, MetaGene Annotator, CD-Hit 454
ViromeScan	Bowtie2	NCBI Genomes: GenBank viral, in-house built reference databases	BMTagger	NCBI human GRCh37/hg19	–	SPFFS	Bash, R, Perl, Java, Bowtie2, Bmtagger, Picard
VirSorter	BLASTp, HMMER3	Pfam, custom	–	–	–	PS	Perl, HMMER3, MCL, MetaGeneAnnotator, MUSCLE, BLAST
VirusFinder	BLAST, BLAT, Bowtie2, BWA	RINS virus DB, or GIB-V	Bowtie2	NCBI human CRCh37/36-hg19/hg18	Trinity	FS(AS/S-pp)	Perl, BLAST+, BLAT, Bowtie2, BWA, iCORN, CREST, GATK, SAMtools, SVDetect, Trinity
VirusHunter	BLAST	NCBI BLAST nt, nr	BLASTn	Host genome	–	PFS(S)	Perl, MySQL, Blast, CD-HIT, RepeatMasker
VirusSeeker	BLAST	NCBI BLAST nt, nr viruses (custom)	BWA-MEM, MegaBlast, BLASTn, BLASTx	NCBI RefSeq: bacteria genomic, NCBI BLAST nt, nr	–	PSF	Perl, SLURM, BLAST, MegaBLAST, BWA-MEM, cutadapt, ea-utils, PRINSEQ, CD-HIT, Tantan, RepeatMasker, Newbler, Phrap
VirusSeq	MOSAIK	GIB-V, hg19 Virus (NCBI human GRCh37/hg19 + TCGA cancer-associated viruses)	MOSAIK	NCBI human GRCh37/hg19	–	FS	Perl, MOSAIK
VirVarSeq	BWA	Custom	–	–	–	SS-pp	BWA, Q-cpileup, R, Fortran, Perl
VMGAP	BLAST, HMMER	NCBI BLAST nt, env_nt, env_nr, NCBI GenBank CDDDB, UniProtDB, OMNIOMEDB, Pfam, TIGRFAM, ACLAME, pfam2gomappingsDB	–	–	–	SSSSS-pp	HMMER, BLAST (NCBI-toolkit), SignalP, TMHMM, PRIAM

*A, assembly; F, filter; P, pre-process; Ph, phylogeny; pp, post-process; S, search. –: not used/specified*.

### Filtering non-target reads

The second step is filtering of non-target, in this case non-viral, reads. Filtering theoretically speeds up subsequent database searches by reducing the number of queries, it helps reduce false positive results and prevents assembly of chimaeric virus-host sequences. However, with lenient homology cutoffs, too many reads may be identified as non-viral, resulting in loss of potential viral target reads. Choice of filtering method depends on the sample type and research goal. For example, with human clinical samples a complete human reference genome is often used, as is the case with SRSA (Isakov et al., 2011), RINS (Bhaduri et al., 2012), VirusHunter (Zhao et al., 2013), MePIC (Takeuchi et al., 2014), Ensemble Assembler (Deng et al., 2015), ViromeScan (Rampelli et al., 2016), and MetaShot (Fosso et al., 2017). Depending on the sample type and expected contaminants, this can be extended to filtering rRNA, mtRNA, mRNA, bacterial or fungal sequences or non-human host genomes. More thorough filtering is displayed by PathSeq (Kostic et al., 2011), SURPI (Naccache et al., 2014), Clinical PathoScope (Byrd et al., 2014), Exhaustive Iterative Assembly (Schürch et al., 2014), VIP (Li et al., 2016), Taxonomer (Flygare et al., 2016), and VirusSeeker (Zhao et al., 2017). PathSeq removes human reads in a series of filtering steps in an attempt to concentrate pathogen-derived data. Clinical PathoScope filters human genomic reads as well as human rRNA reads. Exhaustive Iterative Assembly removes reads from diverse animal species, depending on the sample, to remove non-pathogen reads for different samples. SURPI uses 29 databases to remove different non-targets. VIP includes filtering by first comparing to host and bacterial databases and then to viruses. It only removes reads that are more similar to non-viral references in an attempt to achieve high sensitivity for viruses and potentially reducing false positive results by removing non-viral reads. Taxonomer simultaneously matches reads against human, bacterial, fungal and viral references and attempts to classify all. This only works well on high-performance computing facilities that can handle many concurrent search actions on large data sets. VirusSeeker uses the complete NCBI nucleotide (nt) and non-redundant protein (nr) databases to classify all reads and then filter non-viral reads. Some workflows require a custom, user-provided database for filtering, providing more flexibility but requiring more user-input. This is seen in IMSA (Dimon et al., 2013), VirusHunter (Zhao et al., 2013), VirFind (Ho and Tzanetakis, 2014), and MetLab (Norling et al., 2016), although other workflows may accept custom references as well. In total, 22 workflows filter non-virus reads prior to further analysis (Figure 1B, Table 3). Popular filter tools are read mappers such as Bowtie (Langmead, 2010; Langmead and Salzberg, 2012) and BWA (Li and Durbin, 2009), while specialized software, such as Human Best Match Tagger (BMTagger, NCBI, 2011) or riboPicker (Schmieder, 2011), is less commonly used (Table 2).

**Table 3:** Usability features of classification workflows.

**Workflow**	**PC Platform (Linux, Mac, Windows)**	**Graphical user-interface**	**Freely available**	**User manual**	**Runtime**
Taxonomer	Any (webservice), or Linux, Mac OS	Yes/no (webservice/local installation)	Yes	http://taxonomer.iobio.io/instructions.html	”Real-time, interactive“ - <10 min
Rega Typing Tool v3	Any (webservice)	Yes (webservice)	Yes	http://regatools.med.kuleuven.be/typing/v3/hiv/typingtool/tutorial	500 seqs in 5 h
NBC	Any (webservice)	Yes (webservice)	Yes	http://nbc.ece.drexel.edu/tutorial.php	± 21 h
MePIC	Any (webservice)	Yes (webservice)	Use yes, download upon request	https://mepic.nih.go.jp/mepic/manual/	10 h on 1 CPU, 6 min on 100 CPUs (Megablast only)
Metavir 2	Any (webservice)	Yes (webservice)	Use yes, download no	http://metavir-meb.univ-bpclermont.fr/index.php?page=Tutorial	Hours–days
VirSorter	Any (webservice)	Yes (webservice)	Yes	https://github.com/simroux/VirSorter	Unknown
ClassyFlu	Any (webservice); or Linux, Mac OS	Yes (webservice)	Yes	Supplied with download	Unknown
VIROME	Any (webservice)	Yes (webservice)	Use yes, download no	http://virome.dbi.udel.edu/, Tutorial videos	Unknown
VirFind	Any (webservice)	Yes (webservice)	Use yes, download no	–	±70 h
CaPSID	Linux, Mac OS	Yes	Yes	https://github.com/capsid/capsid/wiki	±20 min
MetLab	Any	Yes	Yes	https://github.com/norling/metlab/blob/master/INSTALL.md	<40 min
MEGAN Community Edition	Any	Yes	Yes	http://ab.inf.uni-tuebingen.de/data/software/megan6/download/manual.pdf	±5.5 h
MEGAN4	Any	Yes	For academic use	http://ab.inf.uni-tuebingen.de/software/megan4/	Unknown
Kraken	Linux	No (Illumina BaseSpace integration?)	Yes	http://ccb.jhu.edu/software/kraken/MANUAL.html	±1 h
FACS	Linux	No	Yes	https://github.com/SciLifeLab/facs	”±20 times faster than BLAT/SSAHA2“
EnsembleAssembler	Linux	No	Yes	https://github.com/xutaodeng/EnsembleAssembler	<5 min (on 8 CPU server)
ViromeScan	Linux, Mac OS	No	Yes	https://sourceforge.net/projects/viromescan/files/?source=navbar	140 sequences/s/CPU
DUDes	Linux	No	Yes	https://sourceforge.net/projects/dudes/files/README.md/download	15–30 min
MetaShot	Linux	No	Yes	https://github.com/bfosso/MetaShot/blob/bfosso-patch-1/MetaShot%20User%20Guide.pdf	2-3x slower than Kraken-MetaPhlAn2
Clinical PathoScope	Any	No	Yes	https://sourceforge.net/p/pathoscope/wiki/clinical_pathoscope/	< 1 h
READSCAN	Linux	No	Yes	http://www.cbrc.kaust.edu.sa/readscan/, Supplied with download - in scripts	<27 min on 16 CPU-HPC - 4 h
Virana	Linux, Mac OS	No	Yes	https://github.com/schelhorn/virana	±30 min/CPU
SURPI	Linux	No	Yes	https://github.com/chiulab/surpi	±1 h (fast), ± 5 h (comprehensive)
RINS	Linux	No	Yes	Supplied with download(?)	±3 h (2CPU), ±15 min (16CPU)
IMSA	Linux, Mac OS	No	Yes	https://sourceforge.net/projects/arron-imsa/files/IMSA_UserManual_v2.pdf/download	hours
GOTTCHA	Linux, Mac OS	No	Yes	https://github.com/LANL-Bioinformatics/GOTTCHA	±4 h (”2-5x slower than Kraken“)
Giant Virus Finder	Linux, Mac OS	No	Yes	http://pitgroup.org/public/giant-virus-finder/latest/README	±30 CPU hours
VIP	Linux (Ubuntu, Biolinux)	No	Yes	https://github.com/keylabivdc/VIP	<2 d
VirusFinder	Linux	No	Yes	https://bioinfo.uth.edu/VirusFinder/VirusFinder-manual.pdf	3 d
ViralFusionSeq	Linux	No	Yes	supplied with download	>1 week
QuasQ	Linux, Mac OS	No	Yes	http://www.statgen.nus.edu.sg/~software/quasq.html	Unknown
IMSA+A	Linux	No	Yes	https://github.com/JeremyCoxBMI/IMSA-A/blob/master/IMSA%2BA_Detailed_Direction.pdf	Unknown
GenSeed-HMM	Linux	No	Yes	https://sourceforge.net/projects/genseedhmm/files	Unknown
VirVarSeq	Linux, Mac OS	No	Yes	https://sourceforge.net/projects/virtools/files/?source=navbar	Unknown
VirusSeeker	Linux	No	Yes	https://wupathlabs.wustl.edu/virusseeker/usage/	Unknown
vFam	Linux, Mac OS	No	Yes	–	Unknown
metaViC	Linux, Mac OS	No	Yes	–	Unknown
PathSeq	Linux, cloud (Amazon EC2, Apache Hadoop)	No	Yes	–	Unknown
Taxy-Pro	Any (webservice or MATLAB)	No	Yes	–	”About three orders of magnitude faster than speed-optimized BLAST“
VirusSeq	Linux, Mac OS	No	Yes	–	>1 week
LMAT	Linux	No	Yes	–	1.3 Mbp/s
RIEMS	Linux	No	Yes	–	10 h (24 CPU-HPC)
SMART	Linux	No	For academic use	https://bitbucket.org/ayl/smart	<10 min or ±2 M reads/min (on HPC - 192 CPUs)
SLIM	Linux, Mac OS	No	Upon request	–	hours of searching, hours for assembly per sample (almost 10x faster than BLAST)
ProViDE	Linux (Ubuntu/Fedora)	No	Academic, non-profit	–	Hours (1 h/100,000 reads - slower than MEGAN)
SRSA	Linux	No	Upon request	–	Unknown
VirusHunter	Linux	No	Non-profit	–	Unknown
Metavir	Any (webservice)	Yes (webservice)	Superceded by newer version	http://metavir-meb.univ-bpclermont.fr/index.php?page=Tutorial	Interactive
VMGAP	?	No	No (only at JCVI)	–	Unknown
”Unknown pathogens from mixed clinical samples“	Windows?	No	No	–	Interactive
Exhaustive Iterative Assembly (Virus Discovery Pipeline)	Linux	No	No	–	Interactive

*Workflows are sorted by: availability of a graphical user-interface (yes-no), runtime (fast-slow), and availability (yes-limited-no). ?: No operating system specified; –: no user manual found*.

### Short read assembly

Prior to classification, the short reads may be assembled into longer contiguous sequences (contigs) and generate consensus sequences by mapping individual reads to these contigs. This helps filter out errors from individual reads, and reduce the amount of data for further analysis. This can be done by mapping reads to a reference, or through so-called *de novo* assembly by linking together reads based on, for instance, overlaps, frequencies and paired-end read information. In viral metagenomics approaches, *de novo* assembly is often the method of choice. Since viruses evolve so rapidly, suitable references are not always available. Furthermore, the short viral genomes generally result in high sequencing coverage, at least for high-titre samples, facilitating *de novo* assembly. However*, de novo* assembly is liable to generate erroneous contigs by linking together reads containing technical errors, such as sequencing (base calling) errors and remaining adapter sequences. Another source of erroneous contigs may be when reads from different organisms in the same sample are similar, resulting in the formation of chimeras. Thus, *de novo* assembly of correct contigs benefits from strict quality control and pre-processing, filtering and taxonomic clustering—i.e., grouping reads according to their respective taxa before assembly. Assembly improvement by taxonomic clustering is exemplified in five workflows: Metavir (Roux et al., 2011), RINS (Bhaduri et al., 2012), VirusFinder (Wang et al., 2013), SURPI (in comprehensive mode) (Naccache et al., 2014), and VIP (Li et al., 2016). Two of the discussed workflows have multiple iterations of assembly and combine algorithms to improve overall assembly: Exhaustive Iterative Assembly (Schürch et al., 2014) and Ensemble Assembler (Deng et al., 2015). In total, 18 of the tools incorporate an assembly step (Figure 1B, Table 4). Some of the more commonly used assembly programs are Velvet (Zerbino and Birney, 2008), Trinity (Grabherr et al., 2011), Newbler (454 Life Sciences), and SPAdes (Bankevich et al., 2012) (Table 2).

**Table 4:** Validation features of classification workflows.

**Workflow**	**Tested by**	**Validation methods**	**Sensitivity (%, no. tests)**	**Specificity (%, no. tests)**	**Precision (%, no. tests)**	**Updates (most recent update)**	**Citations (Google Scholar)**
Kraken	MetaShot, IMSA+A, Taxonomer, GOTTCHA, RIEMS, MetLab	–	67 (21)	92 (6)	97 (2)	Yes (3-3-2016)	334
RINS	CaPSID, Virana, ReadScan, developers	PCR + Sanger sequencing	49 (16)	100 (4)	100 (4)	Yes (10-1-2012)	51
CaPSID	Virana, developers	*in vitro* validation	66 (8)	100 (4)	100 (4)	Yes (2-6-2012)	26
MEGAN 4	MetLab, Bazinet and Cummings, 2012	–	x	x	x	Yes (new version)	752
VirSorter	Developers	Manual curation of prophages	62 (6)	–	90 (6)	Yes (15-2-2017)	34
Virana	Developers	FISH, Southern blot	67 (4)	–	78 (4)	Yes (1-6-2014)	9
vFam	Developers	Compared to previous studies	33 (3)	99 (3)	34 (3)	Yes (9-2-2014)	19
MEGAN Community Edition	IMSA+A	–	x	x	x	Yes (12-7-2017)	22
NBC	MetLab	–	100 (1)	33 (5)	49 (1)	Yes (28-7-2010)	125
SURPI	Taxonomer	–	61 (3)	–	–	Yes (5-6-2015)	128
PathSeq	Readscan, developers	–	51 (10)	–	–	Yes (23-11-20164 m)	158
Metavir 2	ViromeScan	–	82 (1)	–	–	Yes (26-7-2016)	63
Clinical PathoScope	RIEMS	–	18 (13)	–	–	Yes (21-6-2016)	21
ProViDE	MetLab	–	53 (1)	37 (5)	73 (1)	No	19
VirusSeq	–	Serology, colorimetric *in situ* hybridization, immunohistochemistry	–	–	–	Yes (9-8-2013)	50
ViralFusionSeq	–	Sanger sequencing	–	–	–	Yes (19-2-2017)	31
VIP	–	”Independent confirmatory testing results“	–	–	–	Yes (21-2-2017)	5
VirusHunter	–	EM, serology (hemagglutanation inhibition)	–	–	–	Unknown	46
SLIM	–	RT-PCR	–	–	–	Yes^a^	27
”Unknown pathogens from mixed clinical samples"	–	PCR, ELISA	–	–	–	Unknown	1
RIEMS	Developers	–	91 (13)	100 (13)	100 (13)	Yes (10-3-2015)	11
LMAT	Developers	–	50 (6)	–	93 (6)	Yes (17-11-2016)	64
GOTTCHA	Developers	–	71 (1)	–	–	Yes (26-6-2017)	31
IMSA	Developers	–	92 (4)	–	–	Yes (17-4-2014)	10
READSCAN	Developers	–	62 (15)	–	–	No (16-9-2012)	30
FACS	Developers	–	99 (2)	100 (2)	–	Yes (17-12-2015)	39
Taxonomer	Developers	–	95 (4)	91 (1)	–	Yes (3-7-2017)	16
QuasQ	Developers	–	96 (9)	–	99 (9)	Yes (10-7-2014)	5
ViromeScan	Developers	–	100 (1)	–	100 (1)	Yes (29-5-2017)	4
GenSeed-HMM	Developers	–	62 (4)	–	82 (4)	Yes (13-10-2016)	0
IMSA+A	Developers	–	97 (8)	–	81 (8)	Yes (18-7-2017)	0
MetaShot	Developers	–	98 (1)	–	98 (1)	Yes (22-6-2017)	0
SMART	–	–	–	–	–	Yes (19-5-2016)	4
MetLab	–	–	–	–	–	Yes (28-2-2017)	0
EnsembleAssembler	–	–	–	–	–	No (30-11-2014)	41
DUDes	–	–	–	–	–	Yes (22-11-2016)	3
VirusFinder	–	–	–	–	–	Yes (19-6-2014)	49
VirusSeeker	–	–	–	–	–	Yes (21-11-2016)	1
VirVarSeq	–	–	–	–	–	Yes (28-4-2015)	13
Taxy-Pro	–	–	–	–	–	Yes (16-1-2013)	14
VirFind	–	–	–	–	–	Yes (30-6-2017)	31
Metavir	–	–	–	–	–	Yes (new version)	88
metaViC	–	–	–	–	–	Yes (20-6-2017)	NA
MePIC	–	–	–	–	–	Yes	15
ClassyFlu	–	–	–	–	–	Unknown	0
Rega Typing Tool v3	–	–	–	–	–	Unknown	79 + 298
VIROME	–	–	–	–	–	Unknown	59
Giant Virus Finder	–	–	–	–	–	No (7-6-2015)	3
SRSA	–	–	–	–	–	Unknown	40
VMGAP	–	–	–	–	–	Unknown	25
Exhaustive Iterative Assembly (Virus Discovery Pipeline)	–	–	–	–	–	Unknown	11

**Figure 2:**
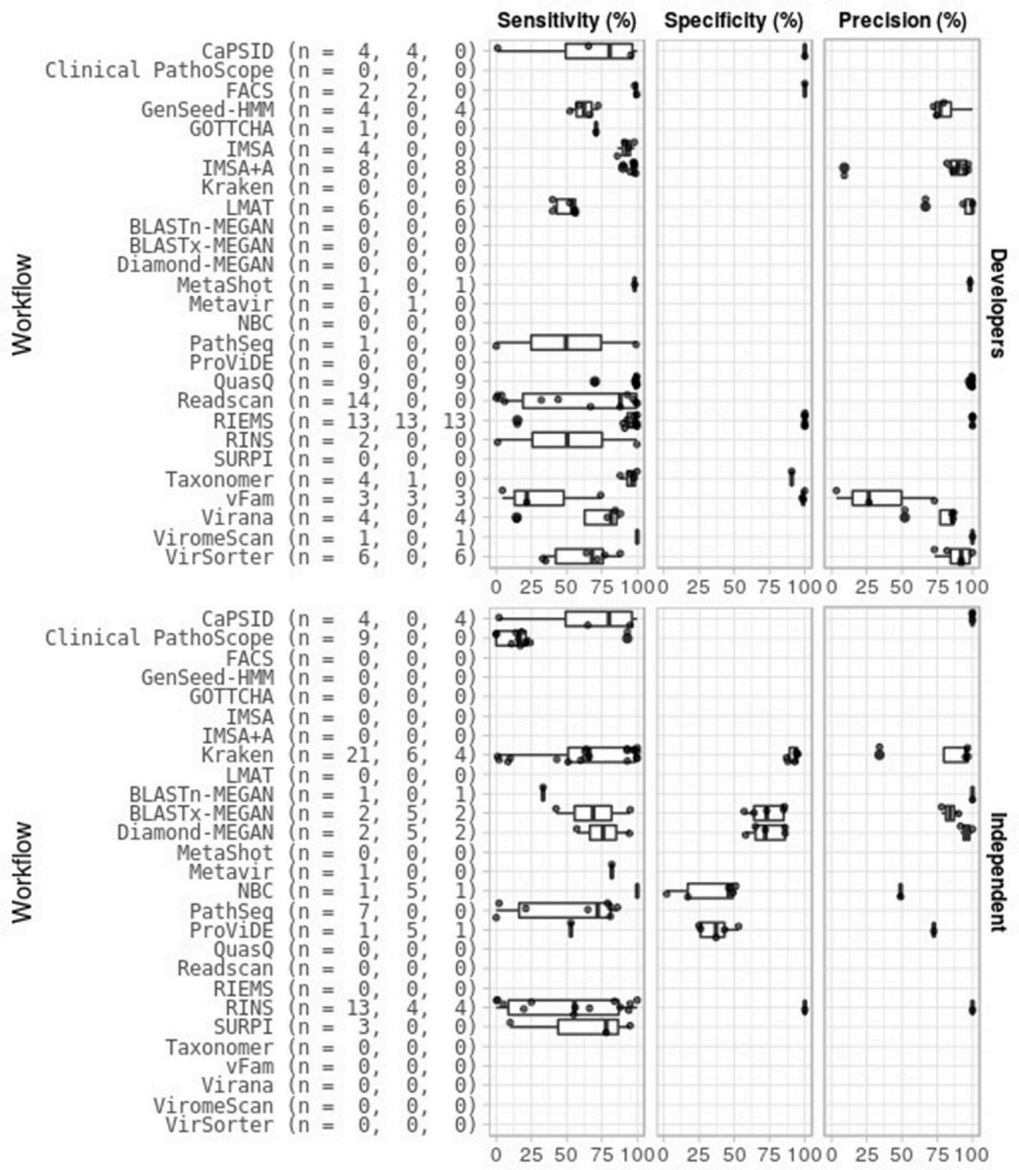
Different benchmark scores of virus classification workflows. Twenty-seven different workflows **(Left)** have been subjected to benchmarks, by the developers **(Top)** or by independent groups **(Bottom)**, measuring sensitivity **(Left column)**, specificity **(Middle column)** and precision **(Right column)** in different numbers of tests. Numbers between brackets (*n* = a, b, c) indicate number of sensitivity, specificity, and precision tests, respectively.

*Workflow were ordered as: Tested by multiple other groups, benchmarked by developers and validated by other experiments, tested by one other group, validated by other experiments, benchmarked by developers, no sign of benchmark tests with updates, no validation and no updates. Tested by: the groups that have tested the workflow. Validation methods: the experiments conducted by the developers to validate the computational results. Sensitivity, specificity and precision: average performance scores of a number (between brackets) of different benchmark tests. Updates: whether or not a pipeline has received updates after publication. Citations: numbers of citations in Google Scholar as of 28 March 2017*.

x: MEGAN visualizes the output of BLAST or DIAMOND and calculates lowest common ancestors. See Figure 2 for different scores.

a *: From personal communication with the developer, we know SLIM has been updated. –: absent/no information available*.

### Database searching

In the search step, sequences (either reads or contigs) are matched to a reference database. Twenty-six of the workflows we found search with the well-known BLAST algorithms BLASTn or BLASTx (Altschul et al., 1990; Table 2). Other often-used programs are Bowtie (Langmead, 2010; Langmead and Salzberg, 2012), BWA (Li and Durbin, 2009), and Diamond (Buchfink et al., 2015). These programs rely on alignments to a reference database and report matched sequences with alignment scores. Bowtie and BWA, which are also popular programs for the filtering step, align nucleotide sequences exclusively. Diamond aligns amino acid sequences and BLAST can do either nucleotides or amino acids. As analysis time can be quite long for large datasets, algorithms have been developed to reduce this time by using alternatives to classical alignment. One approach is to match *k-*mers with a reference, as used in FACS (Stranneheim et al., 2010), LMAT (Ames et al., 2013), Kraken (Wood and Salzberg, 2014), Taxonomer (Flygare et al., 2016), and MetLab (Norling et al., 2016). Exact *k*-mer matching is generally faster than alignment, but requires a lot of computer memory. Another approach is to use probabilistic models of multiple sequence alignments, or profile hidden Markov models (HMMs). For HMM methods, protein domains are used, which allows the detection of more remote homology between query and reference. A popular HMM search program is HMMER (Mistry et al., 2013). ClassyFlu (Van der Auwera et al., 2014) and vFam (Skewes-Cox et al., 2014) rely exclusively on HMM searches, while VMGAP (Lorenzi et al., 2011), Metavir (Roux et al., 2011), VirSorter (Roux et al., 2015), and MetLab can also use HMMER.

All of these search methods are examples of similarity search—homology or alignment-based methods. The other search method is composition search, in which oligonucleotide frequencies or *k*-mer counts are matched to references. Composition search requires the program to be “trained” on reference data and it is not used much in viral genomics. Only two workflows discussed here use composition search: NBC (Rosen et al., 2011) and Metavir 2 (Roux et al., 2014), while Metavir 2 only uses it complementary to similarity search (Data Sheet 1).

All search methods rely on reference databases, such as NCBI GenBank (https://www.ncbi.nlm.nih.gov/genbank/), RefSeq (https://www.ncbi.nlm.nih.gov/refseq/), or BLAST nucleotide (nt) and non-redundant protein (nr) databases (ftp://ftp.ncbi.nlm.nih.gov/blast/db/). Thirty-four workflows use GenBank for their references, most of which select only reference sequences from organisms of interest (Table 2). GenBank has the benefits of being a large, frequently updated database with many different organisms and annotation depends largely on the data providers. Other tools make use of virus-specific databases such as GIB-V (Hirahata et al., 2007) or ViPR (Pickett et al., 2012), which have the advantage of better annotation and curation at the expense of the number of included sequences. Also, protein databases like Pfam (Sonnhammer et al., 1998) and UniProt (UniProt, 2015) are used, which provide a broad range of sequences. Search at the protein level may allow for the detection of more remote homology, which may improve detection of divergent viruses, but non-translated genomic regions are left unused. A last group of workflows requires the user to provide a reference database file. This enables customization of the workflow to the user's research question and requires more effort.

### Post-processing

Classifications of the sequencing reads can be made by setting the parameters of the search algorithm beforehand to return a single annotation per sequence (cut-offs). Another option is to return multiple hits and then determine the relationship between the query sequence and a cluster of similar reference sequences. This process of finding the most likely or best supported taxonomic assignment among a set of references is called post-processing. Post-processing uses phylogenetic or other computational methods such as the lowest common ancestor (LCA) algorithm, as introduced by MEGAN (Huson et al., 2007). Six workflows use phylogeny to place sequences in a phylogenetic tree with homologous reference sequences and thereby classify them. This is especially useful for outbreak tracing to elucidate relationships between samples. Twelve workflows use other computational methods such as the LCA taxonomy-based algorithm to make more confident but less specific classifications (Data Sheet 1). In total, 18 workflows include post-processing (Figure 1B).

### Usability and validation

For broader acceptance and eventual application in a clinical setting, workflows need to be user-friendly and need to be validated. Usability of the workflows varied vastly. Some provide web-services with a graphical user-interface that work fast on any PC, whereas other workflows only work on one operating system, from a command line interface with no user manual. Processing time per sample ranges from minutes to several days (Table 3). Although web-services with a graphical user-interface are very easy to use, such a format requires uploading large GB-sized short read files to a distant server. The speed of upload and the constraint to work with one sample at a time may limit its usability. Diagnostic centers may also have concerns about the security of the data transferred, especially if patient-identifying reads and confidential metadata are included in the transfer. Validation of workflows ranged from high—i.e., tested by several groups, validated by wet-lab experiments, receiving frequent updates and used in many studies—to no evidence of validation (Table 4). Number of citations varied from 0 to 752, with six workflows having received more than 100 citations: MEGAN 4 (752), Kraken, (334), PathSeq (158), SURPI (128), NBC (125), and Rega Typing Tool (377 from two highly cited publications).

### Classification performance

Next, we summarized workflow performance by aggregating benchmark results on simulated viral data from different publications (Figure 2). Twenty-five workflows had been tested for sensitivity, of which 19 more than once. For some workflows, sensitivity varied between 0 and 100, while for others sensitivity was less variable or only single values were available.

For 10 workflows specificities, or true negative rates, were provided. Six workflows had only single scores, all above 75%. The other four had variable specificities between 2 and 95%.

Precision, or positive predictive value was available for sixteen workflows. Seven workflows had only one recorded precision score. Overall, scores were high (>75%), except for IMSA+A (9%), Kraken (34%), NBC (49%), and vFam (3-73%).

Runtimes had been determined or estimated for 36 workflows. Comparison of these outcomes is difficult as different input data were used (for instance varying file sizes, consisting of raw reads or assembled contigs), as well as different computing systems. Thus a crude categorisation was made dividing workflows into three groups that either process a file in a timeframe of minutes (12 workflows: CaPSID, Clinical PathoScope, DUDes, EnsembleAssembler, FACS, Kraken, LMAT, Metavir, MetLab, SMART, Taxonomer and Virana), or hours (19 workflows: Giant Virus Finder, GOTTCHA, IMSA, MEGAN, MePIC, MetaShot, Metavir 2, NBC, ProViDE, Readscan, Rega Typing Tool, RIEMS, RINS, SLIM, SURPI, Taxy-Pro, “Unknown pathogens from mixed clinical samples,” VIP and ViromeScan), or even days (5 workflows: Exhaustive Iterative Assembly, ViralFusionSeq, VirFind, VirusFinder and VirusSeq).

### Correlations between methods, runtime, and performance

**Figure 3:**
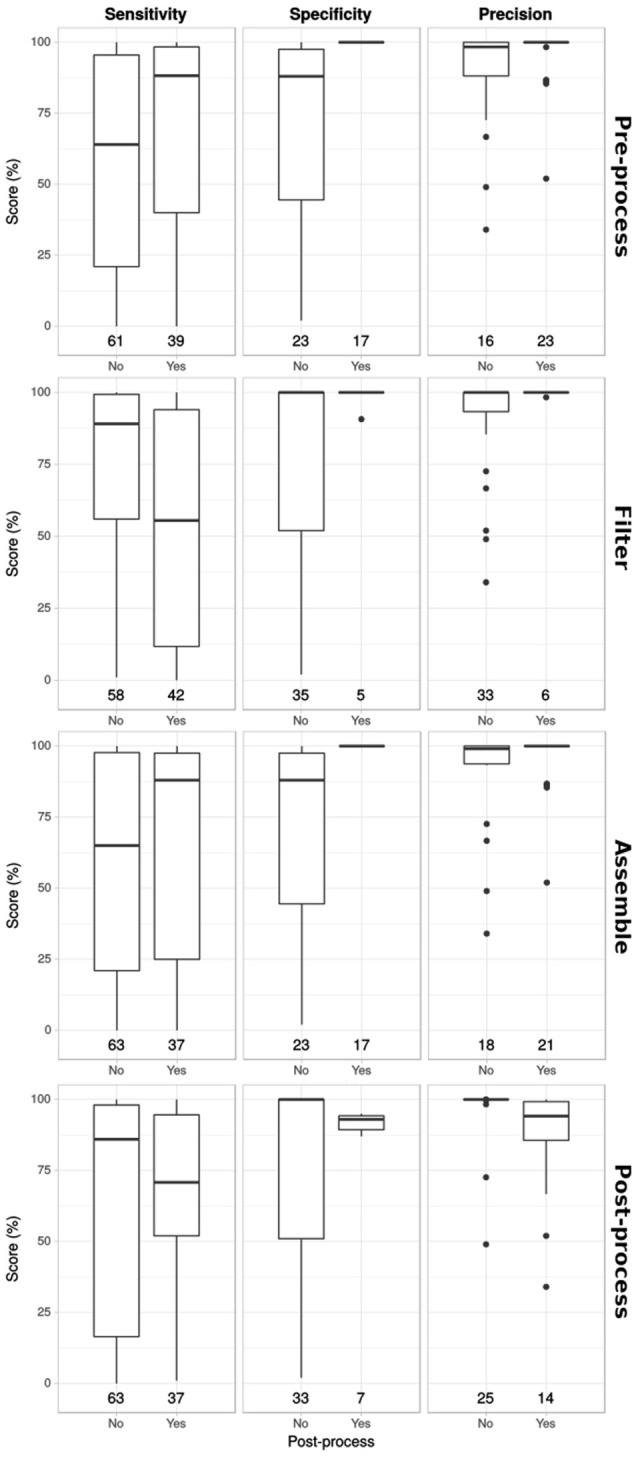
Correlations between performance scores and analysis steps. Sensitivity, specificity and precision scores (in columns) for workflows that incorporated different analysis steps (in rows). Numbers at the bottom indicate number of benchmarks performed.

For 17 workflows for which these data were available, we looked for correlations by plotting performance scores against the analysis steps included (Figure 3). Workflows that included a pre-processing or assembly step scored higher in sensitivity, specificity and precision. Contrastingly, workflows with post-processing on average scored lower on all measures. Pipelines that filter non-viral reads generally had a lower sensitivity and specificity and precision remained high.

**Figure 4:**
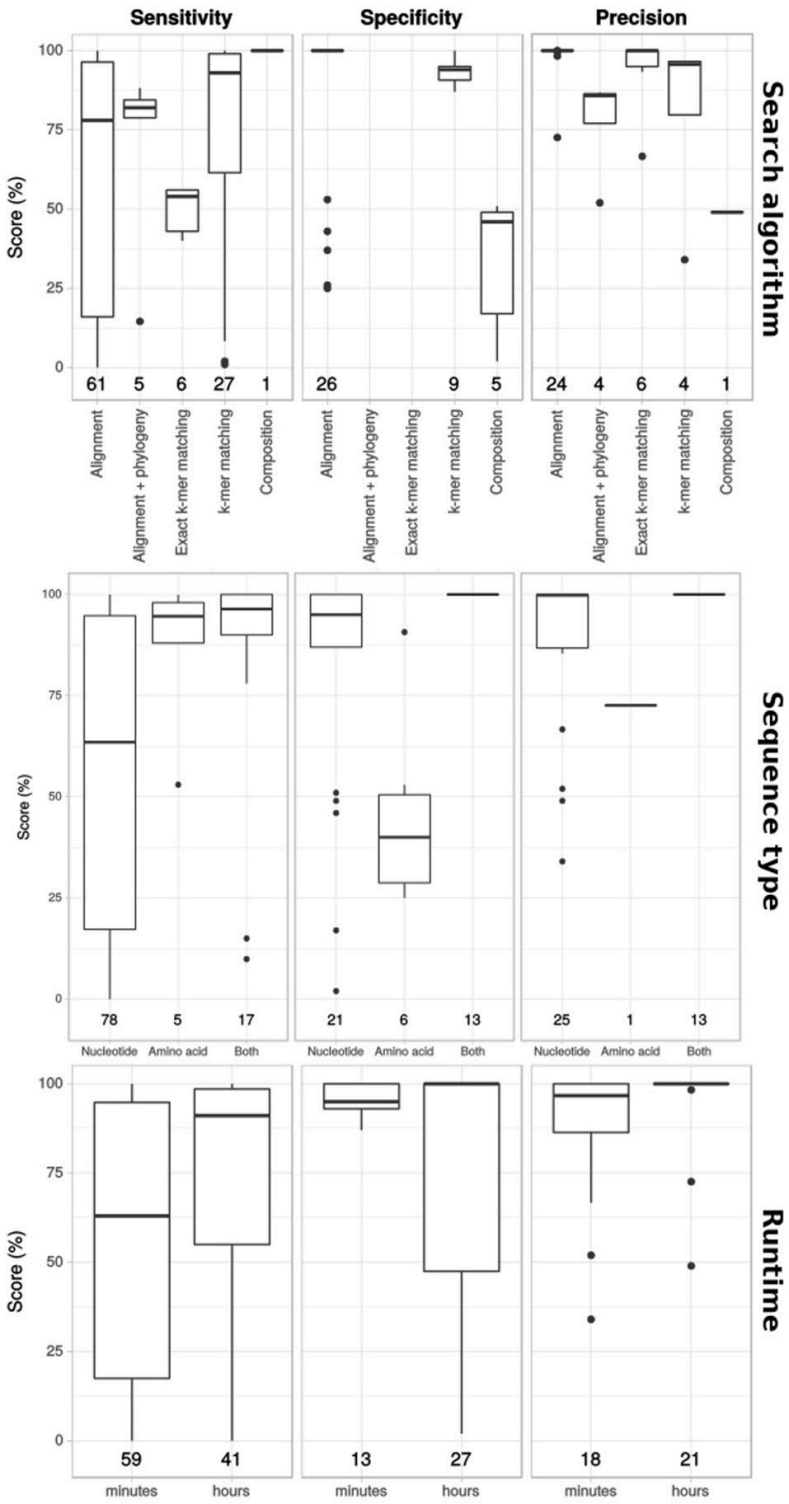
Correlation between performance and search algorithm and runtime. Sensitivity, specificity and precision scores (in columns) for workflows that incorporated different search algorithms, using either nucleotide sequences, amino acid sequences or both, and workflows with different runtimes (rows). Numbers at the bottom indicate number of benchmarks performed.

Next, we visualized correlations between the used search algorithms and the runtime, and the performance scores (Figure 4). Different search algorithms had different performance scores on average. Similarity search methods had lower sensitivity, but higher specificity and precision than composition search. The use of nucleotide vs. amino acid search also affected performance. Amino acid sequences generally led to higher sensitivity and lower specificity and precision scores. Combining nucleotide sequences and amino acid sequences in the analysis seemed to provide the best results. Performance was generally higher for workflows that used more time.

Finally, we inventoried the overall runtime of 17 workflows (Table 5) and separated them based on the inclusion of analysis steps that seemed to affect runtime. This indicated that workflows that included pre-processing, filtering, and similarity search by alignment were more time-consuming than workflows that did not use these analysis steps.

**Table 5:** Correlation between runtime and method.

**Method**	**Minutes**	**Hours**
Pre-process	1	6
No pre-process	7	3
Filter	2	5
No filter	6	4
Assembly	2	3
No assembly	6	6
Nt sequences	6	6
Aa sequences	1	1
Nt + aa sequences	1	2
Alignment	2	8
Alignment + phylogeny	2	0
Exact k-mer matching	3	0
k-mer matching	1	0
Composition search	0	1

*Seventeen workflows, for which runtimes had been reported, were compared to find correlations between runtime and methods. Numbers indicate the number of workflows that process samples in a timeframe of either minutes or hours that use the method listed in the left column. Grayscales are proportional to the total number of scores per group, i.e., like a heatmap lower numbers are lighter and high numbers dark*.

### Applications of workflows

**Figure 5:**
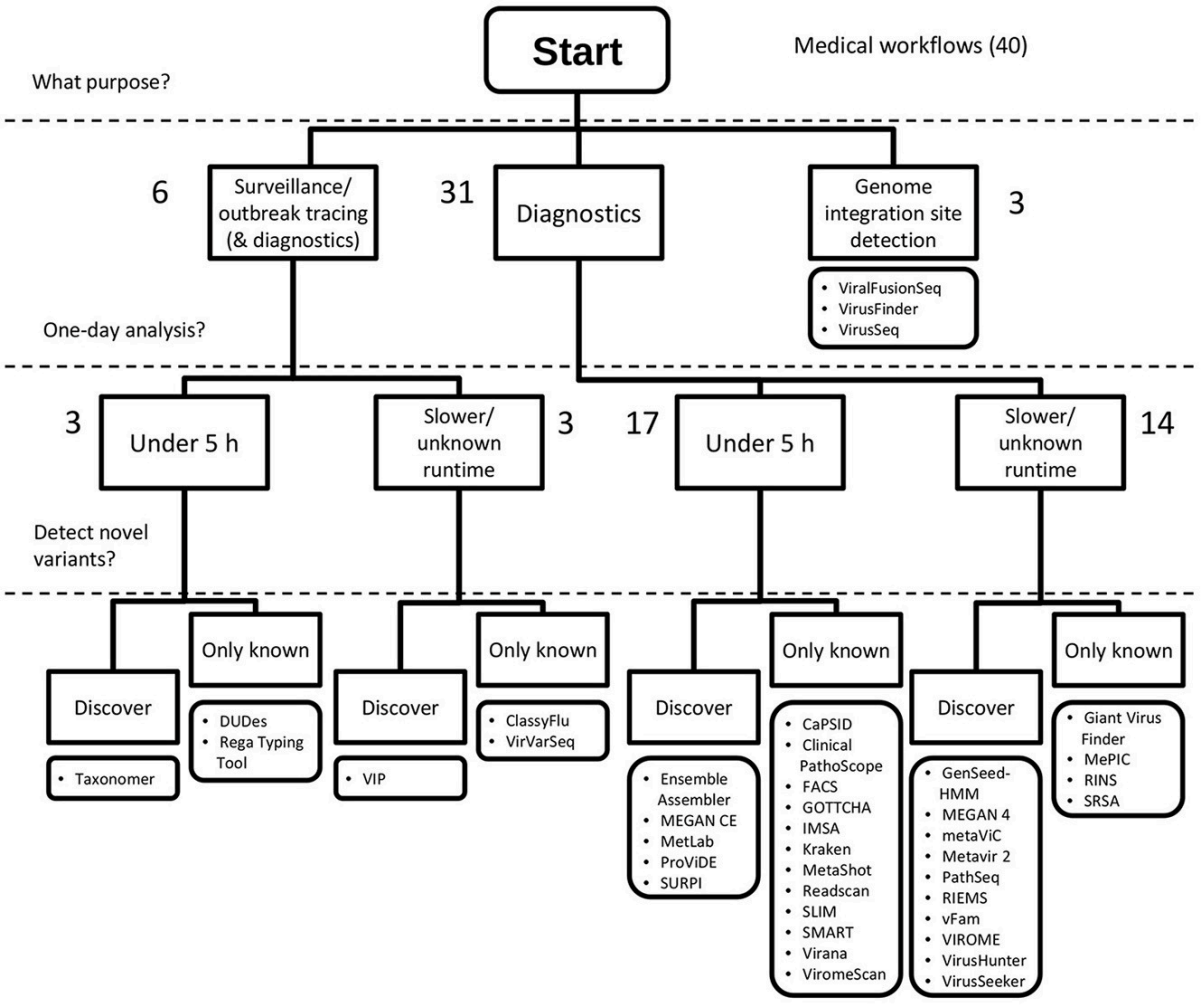
Decision tree for selecting a virus metagenomics classification workflow for medical applications. Workflows are suitable for medical purposes when they can detect pathogenic viruses by classifying sequences to a genus level or further (e.g., species, genotype), or when they detect integration sites. Forty workflows matched these criteria. Workflows can be applied to surveillance or outbreak tracing studies when very specific classification are made, i.e., genotypes, strains or lineages. A 1-day analysis corresponds to being able to analyse a sample within 5 h. Detection of novel variants is made possible by sensitive search methods, amino acid alignment or composition search, and a broad reference database of potential hits. Numbers indicate the number of workflows available on the corresponding branch of the tree.

**Figure 6:**
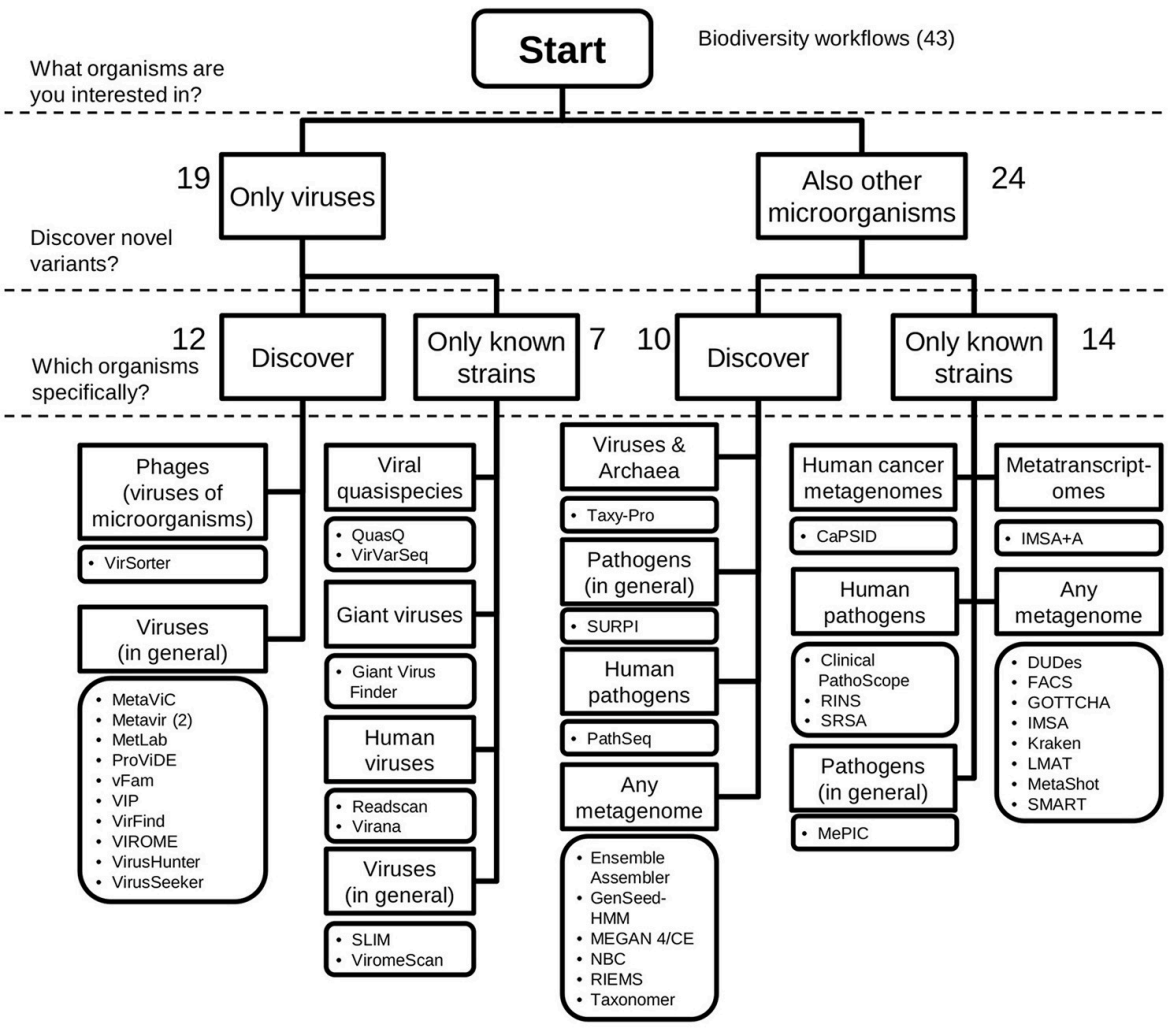
Decision tree for selecting a virus metagenomics classification workflow for biodiversity studies. Workflows for the characterisation of biodiversity of viruses have to classify a range of different viruses, i.e., have multiple reference taxa in the database. Forty-three workflows fitted this requirement. Novel variants can potentially be detected by using more sensitive search methods, amino acid alignment and composition search, and using diverse reference sequences. Finally, workflows are grouped by the taxonomic groups they can classify. Numbers indicate the number of workflows available on the corresponding branch of the tree.

Based on the results of our inventory, decision trees were drafted to address the question of which workflow a virologist could use for medical and environmental studies (Figures 5, 6).

## Discussion

Based on available literature, 49 available virus metagenomics classification workflows were evaluated for their analysis methods and performance and guidelines are provided to select the proper workflow for particular purposes (Figures 5, 6). Only workflows that have been tested with viral data were included, thus leaving out a number of metagenomics workflows that had been tested only on bacterial data, which may be applicable to virus classification as well. Also note that our inclusion criteria leave out most phylogenetic analysis tools, which start from contigs or classifications.

The variety in methods is striking. Although each workflow is designed to provide taxonomic classification, the strategies employed to achieve this differ from simple one-step tools to analyses with five or more steps and creative combinations of algorithms. Clearly, the field has not yet adopted a standard method to facilitate comparison of classification results. Usability varied from a few remarkably user-friendly workflows with easy access online to many command-line programs, which are generally more difficult to use. Comparison of the results of the validation experiments is precarious. Every test is different and if the reader has different study goals than the writers, assessing classification performance is complex.

Due to the variable benchmark tests with different workflows, the data we looked at is inherently limited and heterogeneous. This has left confounding factors in the data, such as test data, references used, algorithms and computing platforms. These factors are the result of the intended use of the workflow, e.g., Clinical PathoScope was developed for clinical use and was not intended or validated for biodiversity studies. Also, benchmarks usually only take one type of data to simulate a particular use case. Therefore, not all benchmark scores are directly comparable and it is impossible to significantly determine correlations and draw firm conclusions.

We do highlight some general findings. For instance, when high sensitivity is required filtering steps should be minimized, as these might accidentally remove viral reads. Furthermore, the choice of search algorithms has an impact on sensitivity. High sensitivity may be required in characterization of environmental biodiversity (Tangherlini et al., 2016) and virus discovery. Additionally, for identification of novel variant viruses and virus discovery *de novo* assembly of genomes is beneficial. Discoveries typically are confirmed by secondary methods, thus reducing the impact in case of lower specificity. For example, RIEMS showed high sensitivity and applies *de novo* assembly. MetLab combines *de novo* assembly with Kraken, which also displayed high sensitivity. When higher specificity is required, in medical settings for example, pre-processing and search methods with the appropriate references are recommended. RIEMS and MetLab are also examples of high-specificity workflows including pre-processing. Studies that require high precision benefit from pre-processing, filtering and assembly. High-precision methods are essential in variant calling analyses for the characterization of viral quasispecies diversity (Posada-Cespedes et al., 2016), and in medical settings for preventing wrong diagnoses. RINS performs pre-processing, filtering and assembly and scored high in precision tests, while Kraken also scored well in precision and with MetLab it can be combined with filtering and assembly as needed.

Clinicians and public health policymakers would be served by taxonomic output accompanied by reliability scores, as is possible with HMM-based search methods and phylogeny with bootstrapping, for example. Reliability scores could also be based on similarity to known pathogens and contig coverage. However, classification to a higher taxonomic rank (e.g., order) is more generally reliable, but less informative than a classification at a lower rank (e.g., species) (Randle-Boggis et al., 2016). Therefore, the use of reliability scores and the associated trade-offs need to be properly addressed per application.

Besides, medical applications may be better served by a functional rather than a taxonomic annotation. For example, a clinician would probably find more use in a report of known pathogenicity markers than a report of species composition. Bacterial metagenomics analyses often include this, but it is hardly applied to virus metagenomics. Although valuable, functional annotation further complicates the analysis (Lindgreen et al., 2016).

Numerous challenges remain in analyzing viral metagenomes. First is the problem of sensitivity and false positive detections. Some viruses that exist in a patient may not be detected by sequencing, or viruses that are not present may be detected because of homology to other viruses, wrong annotation in databases or sample cross-contamination. These might both lead to wrong diagnoses. Second, viruses are notorious for their recombination rate and horizontal gene transfer or reassortment of genomic segments. These may be important for certain analyses and may be handled by bioinformatics software. For instance, Rega Typing Tool and QuasQ include methods for detecting recombination. Since these events usually happen within species and most classification workflows do not go deeper into the taxonomy than the species level, this is something that has to be addressed in further analysis. Therefore, recombination should not affect the results of the reviewed workflows much. Further information about the challenges of analyzing metagenomes can be found in Edwards and Rohwer (2005); Wommack et al. (2008); Wooley and Ye (2009); Tang and Chiu (2010); Wooley et al. (2010); Fancello et al. (2012); Thomas et al. (2012); Pallen (2014); Hall et al. (2015); Rose et al. (2016); McIntyre et al. (2017), and Nieuwenhuijse and Koopmans (2017).

An important step in the much awaited standardization in viral metagenomics (Fancello et al., 2012; Posada-Cespedes et al., 2016; Rose et al., 2016), necessary to bring metagenomics to the clinic, is the possibility to compare and validate results between labs. This requires standardized terminology and study aims across publications, which enables medically oriented reviews that assess suitability for diagnostics and outbreak source tracing. Examples of such application-focused reviews can be found in the environmental biodiversity studies (Oulas et al., 2015; Posada-Cespedes et al., 2016; Tangherlini et al., 2016). Reviews then provide directions for establishing best practices by pointing out which algorithms perform best in reproducible tests. For proper comparison, metadata such as sample preparation method and sequencing technology should always be included—and ideally standardized. Besides, true and false positive and negative results of synthetic tests have to be provided to compare between benchmarks.

Optimal strategies for particular goals should then be integrated in a user-friendly and flexible software framework that enables easy analysis and continuous benchmarking to evaluate current and new methods. The evaluation should include complete workflow comparisons and comparisons of individual analysis steps. For example, benchmarks should be done to assess the addition of a *de novo* assembly step to the workflow and measure the change in sensitivity, specificity, etc. Additionally, it remains interesting to know which assembler works best for specific use cases as has been tested by several groups (Treangen et al., 2013; Scholz et al., 2014; Smits et al., 2014; Vázquez-Castellanos et al., 2014; Deng et al., 2015). The flexible framework should then facilitate easy swapping of these steps, so that users can always use the best possible workflow. Finally, it is important to keep reference databases up-to-date by sharing new classified sequences, for instance by uploading to GenBank.

All these steps toward standardization benefit from implementation of a common way to report results, or minimum set of metadata, such as the MIxS by the genomic standard consortium (Yilmaz et al., 2011). Currently several projects exist that aim to advance the field to wider acceptance by validating methods and sharing information, e.g., the CAMI challenge (http://cami-challenge.org/), OMICtools (Henry et al., 2014), and COMPARE (http://www.compare-europe.eu/). We anticipate steady development and validation of genomics techniques to enable clinical application and international collaborations in the near future.

## Author contributions

AK and MK conceived the study. SN designed the experiments and carried out the research. AK, DS, and HV contributed to the design of the analyses. SN prepared the draft manuscript. All authors were involved in discussions on the manuscript and revision and have agreed to the final content.

### Conflict of interest statement

The authors declare that the research was conducted in the absence of any commercial or financial relationships that could be construed as a potential conflict of interest.
